# Exploring treatment with Ribociclib alone or in sequence/combination with Everolimus in ER^+^HER2^−^Rb wild-type and knock-down in breast cancer cell lines

**DOI:** 10.1186/s12885-020-07619-1

**Published:** 2020-11-19

**Authors:** Oliviero Marinelli, Emanuela Romagnoli, Federica Maggi, Massimo Nabissi, Consuelo Amantini, Maria Beatrice Morelli, Matteo Santoni, Nicola Battelli, Giorgio Santoni

**Affiliations:** 1 School of Pharmacy, University of Camerino, 62032 Camerino, MC Italy; 2 Medical Oncology Unit, Hospital of Macerata, Macerata, Italy; 3 Department of Molecular Medicine, University of Rome Sapienza, Rome, Italy; 4 School of Bioscience and Veterinary Medicine, University of Camerino, Camerino, MC Italy

**Keywords:** Breast cancer, ER + HER2-, CDK4/6 inhibitor, Ribociclib, Everolimus, Rb

## Abstract

**Background:**

Breast cancer (BC) is the second most common type of cancer worldwide. Among targeted therapies for Hormone Receptor-positive (HR^+^) and Human Epidermal growth factor Receptor 2-negative (HER2^−^) BC, the Cyclin-Dependent Kinases (CDK4/6) are targeted by inhibitors such as Ribociclib (Rib); however, resistance to CDK4/6 inhibitors frequently develops. The aim of this work is to assess in vitro activity of Rib and Everolimus (Eve) in HR^+^HER2^−^ MCF-7 and HR^−^HER2^−^BT-549 BC cell lines.

**Methods:**

HR^+^HER2^−^ MCF-7 and HR^−^HER2^−^ BT-549 BC cell lines were treated with increasing concentration of Rib and Eve (up to 80 μg/mL) for 48–72 h. Subsequently, HR^+^HER2^−^ MCF-7 cells were silenced for Retinoblastoma (Rb) gene, and thus, the effect of Rib in sequential or concurrent schedule with Eve for the treatment of both Rb wild type or Rb knock-down MCF-7 in vitro was evaluated. Cell viability of HR^+^HER2^−^ MCF-7cells treated with sequential and concurrent dosing schedule was analyzed by MTT assay. Moreover, cell cycle phases, cell death and senescence were evaluated by cytofluorimetric analysis after treatment with Rib or Eve alone or in combination.

**Results:**

The sequential treatment didn’t produce a significant increase of cytotoxicity, compared to Rib alone. Instead, the cotreatment synergized to increase the cytotoxicity compared to Rib alone. The cotreatment reduced the percentage of cells in S and G2/M phases and induced apoptosis. Rib triggered senescence and Eve completely reversed this effect in Rb wild type BC cells. Rib also showed Rb-independent effects as shown by results in Rb knock-down MCF-7.

**Conclusion:**

Overall, the Rib/Eve concurrent therapy augmented the in vitro cytotoxic effect, compared to Rib/Eve sequential therapy or single treatments.

**Supplementary Information:**

The online version contains supplementary material available at 10.1186/s12885-020-07619-1.

## Background

Molecular profiling of breast cancers (BC) has identified several intrinsic subtypes. The majority of estrogen receptor positive (ER^+^) BC are classified as either luminal A or B. Luminal A tumors are typically more sensitive to therapy, while luminal B tumors show a more aggressive and endocrine-resistant phenotype. The endocrine therapies, which target ER activity, are standard treatments for patients with ER^+^ and human epidermal growth factor receptor negative (HER2^−^) BC in both the early and the advanced/metastatic stages [1, 2].

Recent advances in elucidating the molecular mechanisms of crosstalk among ER, cell-cycle regulating proteins and intracellular signaling pathways, have provided the rationale for combining endocrine therapies with targeted agents [3]. Dysregulated cellular proliferation, one of the hallmarks of cancer, is mediated by aberrant activation of the cell cycle machinery through the biological effects of cyclin-dependent kinases (CDKs) [4]. The generation of non-selective CDK inhibitors failed due to combined lack of efficacy and excessive toxicity reported by clinical trials across different cancer types [5].

The clinical development of second generation of CDK4/6-selective inhibitors, namely Ribociclib (LEE011), Palbociclib and Abemaciclib, has completely changed the prognosis of patients with hormone receptor positive HR^+^HER2^−^ BC [6, 7]. Ribociclib (Rib) is a selective, orally bioavailable, small molecule designed to competitively bind to the ATP-binding pockets of CDK4/6 [8], blocking the phosphorylation of the retinoblastoma protein (pRb), thereby preventing cell cycle progression and inducing G1 phase arrest [2, 9, 10]. The CDK4/6 and cyclin D1 are part of the cyclin D/CDK4/6/Rb/E2F1 pathway controlling the cell cycle progression. CDK4/6 overexpression and CCND1 amplification are frequently detected in HR^+^ BC [9, 11]; in addition, Rb inactivation, E2F1 overexpression and the persistent cyclin D1 expression are frequently associated with the development of endocrine resistance in HR^+^ BC [12].

Preclinical and multiple trials regarding Rib administration are ongoing across different tumor types including BRAFv600 and NRAS-mutant melanomas, non-small-cell-lung carcinoma, gynecologic cancers such as cervical cancers, neuroblastoma, nasopharyngeal carcinoma, neck squamous cell carcinoma, thyroid cancers and lymphomas [13–18]. Rib received FDA approval in 2016, in combination with letrozole for the first-line treatment of HR^+^ and HER2^−^ advanced breast cancer (ABC) [2, 9]. In preclinical study it demonstrated inhibitory activity predominantly against ER^+^ cell lines, suggesting that ER^+^BC might be particularly susceptible to CDK4/6 inhibition [9]. Although single agent activity has been demonstrated for Rib, it has also been shown to enhance the activity of combination partners and delay the development of resistance in preclinical and in clinical studies. Thus, combination of Rib with the PI3K inhibitor, Alpelisib, resulted in enhanced tumor regression, increased response rates and progression free survival, compared with the single agents alone [9, 10]. Moreover, in grade II/III HR^+^HER2^−^ invasive BC, it was evidenced an enhanced reduction in cell proliferation upon combination of Rib plus letrozole, compared to letrozole alone [9].

The PI3K/Akt/mTOR pathway regulates several intracellular processes including cancer cell growth, survival, and proliferation. Its increased activation is associated with resistance to endocrine therapy. Mammalian target of rapamycin (mTOR) is a key regulator of these signals functioning in two intracellular complexes: mTOR complex 1 (mTORC1) and mTOR complex 2 (mTORC2). Everolimus (Eve) is an mTOR inhibitor that arrests the cells at both G1 or G2/M phases in a dose-dependent manner [19] and prevents the downstream signaling required for cell cycle progression, cell growth, and proliferation [20]. Administered as single agent, daily or weekly, Eve shows modest clinical activity in patients with metastatic BC, while promising results are obtained combing Eve with endocrine therapy [20] and CDK4/6 inhibitors [21].

On this scenario, we assessed the in vitro activity of Rib in HR^+^HER2^−^ MCF-7 and HR^−^HER2^−^ BT-549 BC cell line. Furthermore, we focused on the sequential or concurrent effect of Rib and Eve in models Rb wild type (WT) and Rb knockdown (KD) MCF-7 cells.

## Methods

### Cell culture

The MCF-7 BC (HR^+^, HER2^−^) and BT-549 (HR^−^, HER2^−^) cell lines (purchased from ATCC, LGC Standards, Milan, Italy) were cultured in RPMI 1640 medium (Lonza, Milan, Italy) supplemented with 10% foetal bovine serum (FBS), 2 mM L-glutamine, 100 IU/ml penicillin and 100 μg streptomycin. Cell line was maintained at 37 °C with 5% CO_2_ and 95% humidity. BT-449 were used because they harbor a Rb mutation (homozygous for Rb1 c265_607del343).

### Compounds

Rib and Eve were furnished by Novartis Pharma AG (Basel, Swizterland), resuspended in DMSO and 20 mg/ml (for Rib) and 8 mg/ml (for Eve) aliquots were prepared and stored at − 20 °C. Each aliquot was used one time.

### Rb silencing

Small interfering RNAs (siRNAs) targeted Rb (hs.Rb.RB1.13.1) was purchased from Integrated DNA Technologies (Leuven, Belgium). MCF-7 cells were plated at a density of 1 × 10^5^ cells/ml. After overnight incubation, transfections were achieved with 7.5 μl/ml of the reagent TransIT-X2 (Mirus Bio, Madison, WI, USA) and 10 nM of hs.Rb.RB1.13.1 and TransIT-X2 used as negative control according to the manufacturer’s instructions. The cells were harvested at 72 h post-transfection for analysis. The efficiency of transfection was evaluated by western blot analysis.

### Western blot analysis

Cells were lysed in lysis buffer containing protease inhibitor cocktail (Sigma-Aldrich, Milan, Italy). Lysates were separated on 6% SDS polyacrylamide gel and transferred onto Hybond-C extra membranes (GE Healthcare, Chicago, IL, USA). Membrane was blocked overnight at 4 °C with 5% milk and 3% BSA. Then, membrane was incubated 1 h at room temperature with mouse anti-human Rb (1:300, Santa Cruz Biotechnologies, Dallas, TX, USA) antibody (Ab) and overnight with mouse anti-β-actin Ab (1:3000, Santa Cruz Biotechnologies), followed by the incubation (room temperature, 1 h) with HRP-conjugated anti-mouse secondary Ab (Cell Signaling Technology, Milan, Italy). Peroxidase activity was visualized with the LiteAblot® PLUS and LiteAblot® TURBO (EuroClone, Milan, Italy) kits and densitometric analysis. The expression of Rb was evaluated both in Rb WT and KD MCF-7 cells.

### MTT assay

Twenty thousand cells/ml of Rb WT and Rb KD MCF-7 and BT-549 cells was seeded before drug treatment in 96-well plates. After 1 day of incubation, compounds or vehicles were added. Six replicates were used for each treatment. At the indicated time points, cell viability was assessed by adding 0.8 mg/ml of MTT (Sigma-Aldrich) to the media. After 3 h, the plates were centrifuged, the supernatant was discharged, and the pellet was solubilized with 100 μl/well DMSO. The absorbance of the samples against a background control (medium alone) was measured at 570 nm using an ELISA reader microliter plate (BioTek Instruments, Winooski, VT, USA). The statistical analysis of IC50 levels for each single treatment was performed using Prism 5.0a (Graph Pad).

In the sequential schedule experiments where both Rib and Eve were used, the Rb WT and KD MCF-7 cell lines were treated with the first compound for 72 h, then the cells were washed out and treated with the second compound for additional 48 h.

Additionally, cells were treated with combination of both compounds (concurrent therapy) at (5, 10 and 20 μg/ml) for 72 h. Synergistic activity of the Rib/Eve combination was determined by the isobologram and combination index (CI) methods (CompuSyn Software, ComboSyn, Inc. Paramus, NJ 2007). The CI was used to express synergism (CI < 1), additivity (CI = 1) or antagonism (CI > 1) and was calculated according to the standard isobologram equation [22].

### Cell cycle analysis

Two × 10^4^ Rb WT and KD MCF-7cells/ml were treated with the appropriate drugs, collected and fixed in 70% ethanol and then washed with staining buffer (PBS, 2% FBS and 0.01% NaN_3_). Next, the cells were treated with 100 μg/ml ribonuclease A solution, incubated for 30 min at 37 °C, stained for 30 min at room temperature with PI 20 μg/ml and then analysed by flow cytometry using linear amplification.

### Annexin/PI staining

Cell death was evaluated using Annexin V-FITC and PI staining followed by flow cytometry and FACS analysis. Two × 10^4^/ml Rb WT and KD MCF-7cells were treated with vehicle, Rib and/or Eve n in combination at 10/15 μg/ml, 1:1.5 ratio for up to 72 h. After treatment, cells were stained with 5 μl of Annexin V-FITC (eBioscience) and 20 μg/ml PI for 10 min at room temperature and washed once with binding buffer (10 mM Hepes/NaOH pH 7.4, 140 mM NaCl, 2.5 mM CaCl2). The percentage of positive cells determined over 10,000 events was analyzed using flowcytometry and CellQuest software.

### Senescence analysis

Senescence analysis was performed by flow cytometry and FACS analysis to evaluate the senescence-associated β-galactosidase activity. Cells were treated with Rib/Eve in combination at 10/15 μg/ml, 1:1.5 ratio and Rib or Eve alone. The senescence assay was performed using the fluorogenic substrate C12FDG. Cells were incubated for 1 h at 37 °C and 5% CO2 with 100 nM bafilomycin A1 in culture medium to induce lysosomal alkalization at pH 6 and, then, for 1 h with 33 μM C12FDG. Samples were immediately analyzed using FACScan cytofluorimeter using the CellQuest software. The C12-fluorescein signal was measured on the FL-1 detector, and β-galactosidase activity was estimated as percentage of positive cells [23].

### Statistical analysis

The data presented represent the mean and standard deviation (SE) of at least 3 independent experiments. The statistical significance was determined by analysis of variance or Student’s t-test, *p < 0.01 or by ANOVA with Bonferroni’s post-test.

## Results

### Expression of Rb in MCF-7 cell line

Rib targets the CDK4/6Rb/E2F1 pathway in ER^+^ HER2^−^ BCs. In order to evaluate the Rb-dependent and independent Rib effects in MCF-7 BC cell line, we down-regulated the Rb gene expression by RNA interfering. The efficiency of Rb silencing, at 72 h post transfection was analysed by western blot analysis using an anti-human Rb Ab. MCF-7 cells showed high Rb protein levels (Rb WT MCF-7), while a marked reduction of Rb protein (about 80%) was evidenced by densitometric analysis in Rb silenced (Rb KD) MCF-7 cells (Fig. S1).

### Rib and eve inhibited cell viability of Rb WT and KD MCF-7 cells in a time- and dose-dependent manner

**Fig. 1:**
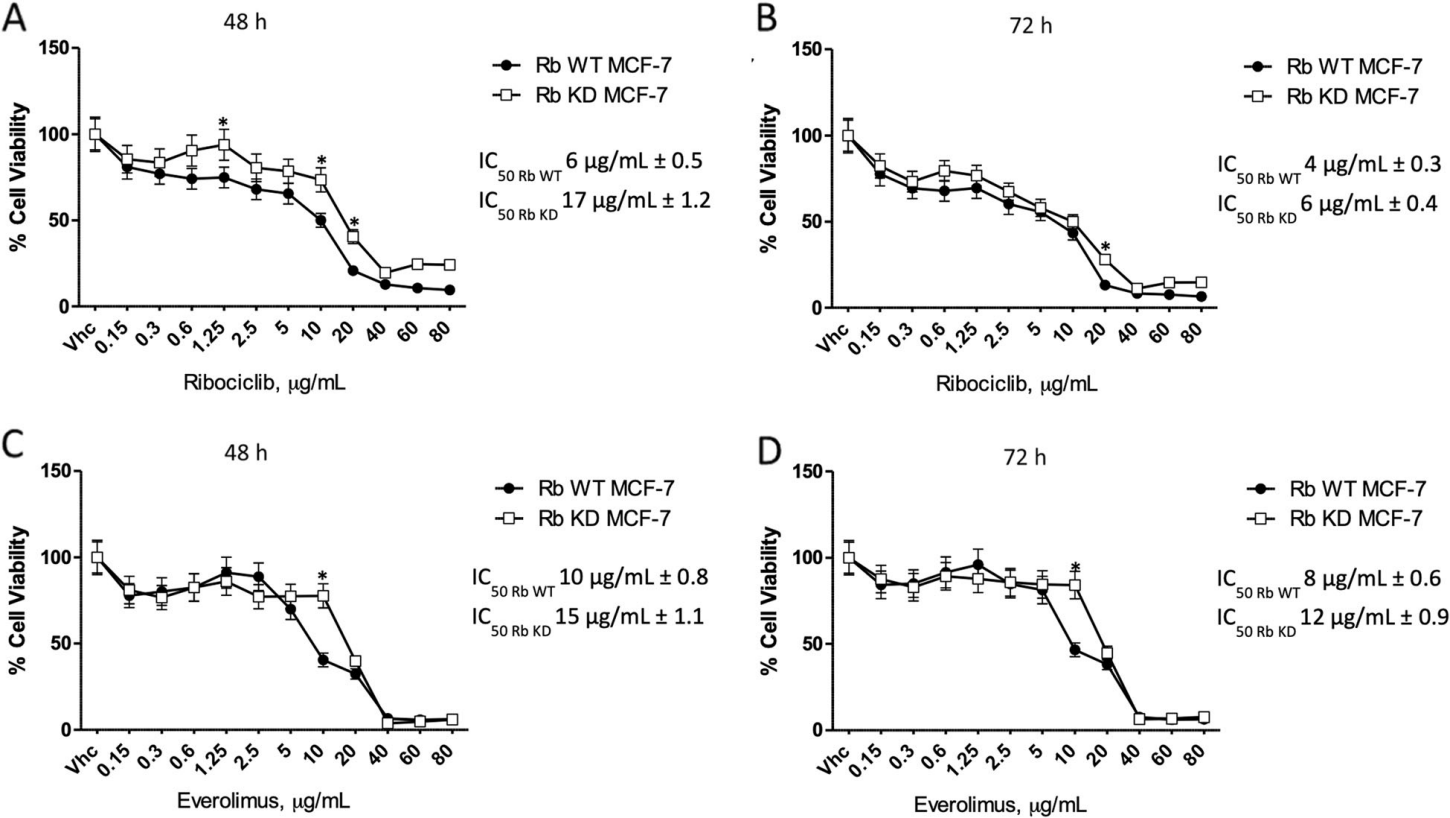
Rib and Eve induced cytotoxicity in Rb WT and Rb KD MCF-7 cell lines. Cell viability was determined by MTT assay. Rb WT MCF-7 and Rb KD MCF-7 cells were treated for **a** 48 h and **b** 72 h with different concentrations of Ribociclib; **c** 48 h and **d** 72 h with different concentrations of Everolimus. Data shown are expressed as mean ± SE of three separate experiments. *p < 0.05 Rb WT MCF-7 vs Rb KD MCF-7

The effect of Rib or Eve as single treatment on Rb WT and KD MCF-7 cell viability, was evaluated by MTT assay at 48 and 72 h after treatment. Cells were treated with different doses of Rib or Eve (from 0.1 to 80 μg/ml). The results indicate that both Rib and Eve, alone, markedly inhibited the viability of Rb WT MCF-7 cells (Rib: IC_50_ at 48 and 72 h: 6 ± 0.5 and 4 ± 0.3 μg/ml (Fig. 1a and b), respectively; Eve: IC_50_ at 48 and 72 h: 10 ± 0.8 μg/ml and 8 ± 0.6 μg/ml (Fig. 1c and d), respectively). Furthermore, in Rb KD MCF-7 cells, the IC_50_ of Rib at 48 and 72 h after treatment was 17 ± 1.2 μg/ml and 6 ± 0.4 μg/ml (Fig. 1a and b) respectively; likewise, for Eve the IC_50_ was 15 ± 1.1 μg/ml and 12 ± 0.9 μg/ml (Fig. 1c and d). Moreover, the effect of Rib at different concentrations (up to 80 μg/ml) and times (48–72 h) was also evaluated by MTT assay in the triple-negative BT-549 cell line [24]. The IC_50_ of Rib against BT-549 breast cancer cell line, at 48–72 h was 3.2 and 12.7 μg/ml, respectively (Fig. S2).

### Effect of sequential dosing schedule of rib and eve in Rb WT and KD MCF-7 cells

Based on the different mechanism of action of Rib and Eve, we hypothesized that the use of different sequential dosing schedules may enhance the effects of these drugs, compared with the single treatment. Thus, effect of sequential dosing schedule with Rib for 72 h followed by Eve for further 48 h and vice versa was evaluated both in Rb WT and KD MCF-7 cells (Table 1A).

**Table 1:** Sequential or concurrent dosing schedule in Rb WT and KD MCF-7 cells

**A) Sequential dosing schedule**		
	3 days	2 days
1	Ribociclib	Vehicle
2	Ribociclib	Everolimus
3	Everolimus	Vehicle
4	Everolimus	Ribociclib
**B) Concurrent dosing schedule**		
	3 days	
1	Ribociclib + Everolimus	
2	Ribociclib + Vehicle	
3	Everolimus + Vehicle	

A) Sequential dosing schedule of combination of Rib and Eve; B) Concurrent dosing schedule of combination of Rib and Eve

**Fig. 2:**
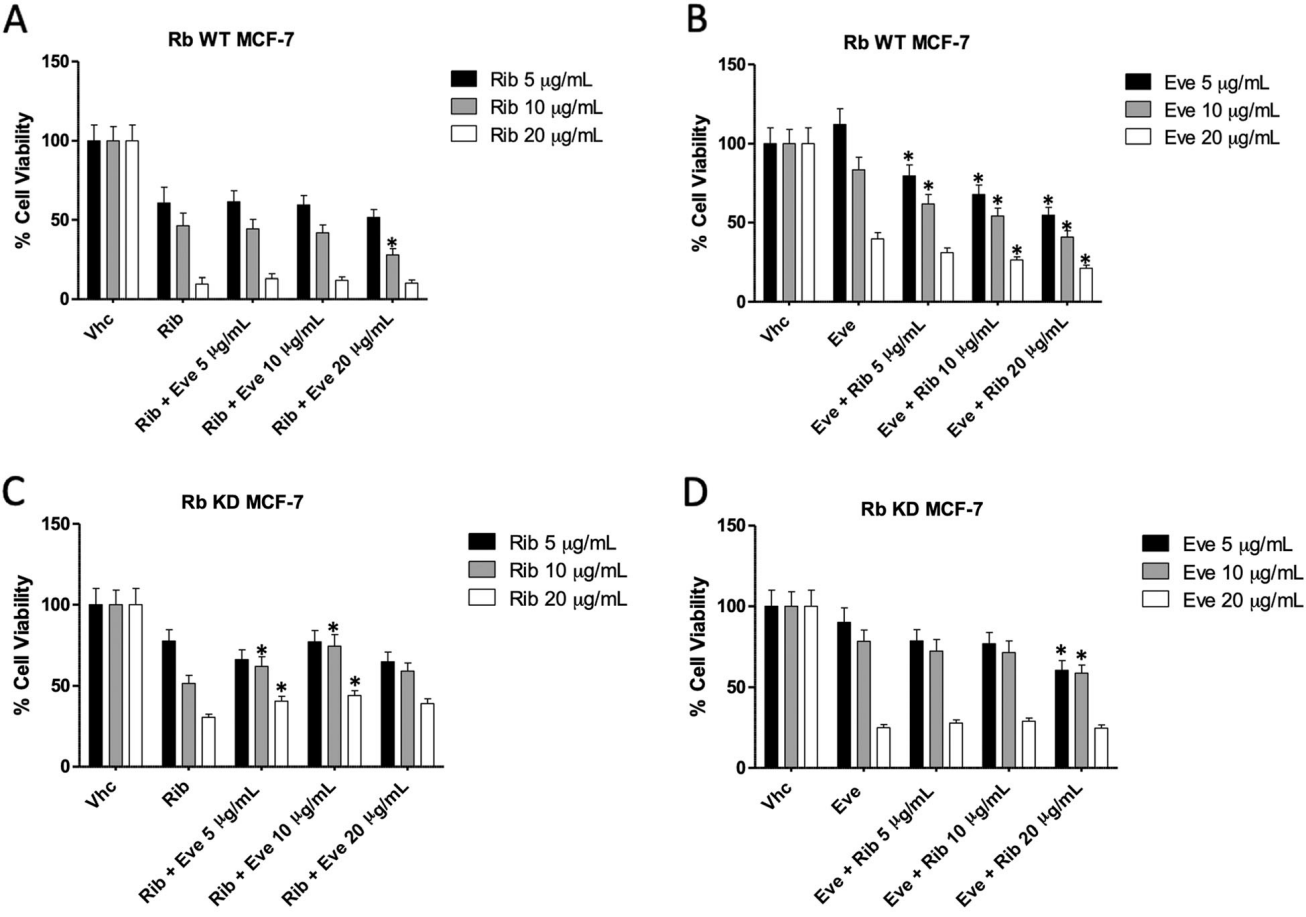
Sequential administration of Rib and Eve induced cytotoxicity in Rb WT and KD MCF-7 cells. Cell viability was determined by MTT assay. Rb WT and KD MCF-7 cells were treated with different dosing schedule: **a**-**c** Rib 3 days followed by Eve 2 days and **b**-**d** Eve 3 days followed by Rib 2 days with different concentrations (5, 10 and 20 μg/mL). Data shown are expressed as mean ± SE of three separate experiments. *p < 0.05 Sequential schedule vs single drug

Rib and Eve were tested at doses of 5, 10 and 20 μg/ml. Rb WT and KD MCF-7 cells treated with Rib or Eve alone, washed out at 72 h and incubated for additional 48 h in medium without drugs, was used as control. No major differences were observed at any Rib/Eve combinations both in Rb WT and KD MCF-7 cells, except for Rib/Eve at 10/20 μg/ml in Rb WT MCF-7 cells (Fig. 2a and c). On the contrary, the sequential administration of Eve at each dose, followed by Rib, especially at low doses (5 and 10 μg/ml), markedly increased the Eve-mediated cytotoxic effects in Rb WT MCF-7 cells (Fig. 2b). No major effect was observed in Rb KD MCF-7 cells, except for Eve followed by Rib 20 μg/ml (Fig. 2d).

### Effect of concurrent dosing schedule of rib and eve in Rb WT and KD MCF-7 cells

**Fig. 3:**
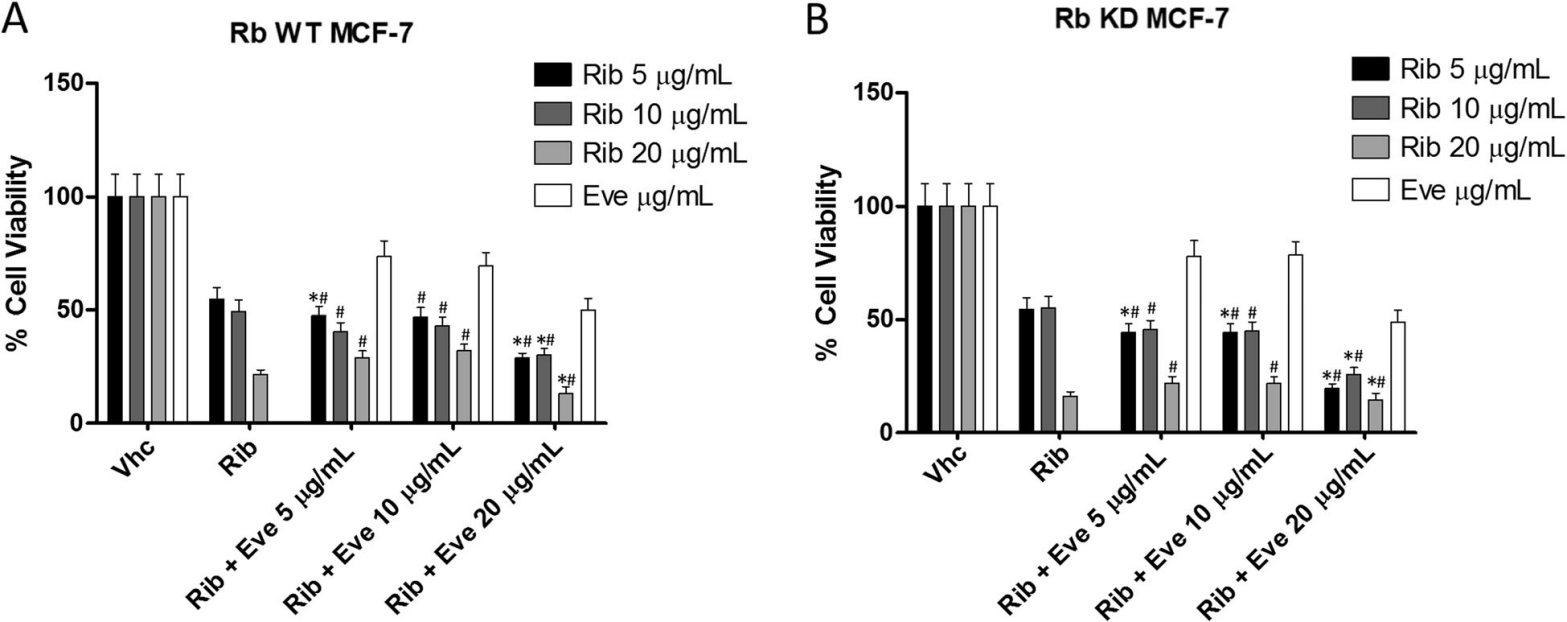
Concurrent administration of Rib and Eve induced cytotoxicity in Rb WT and KD MCF-7 cells.Cell viability was determined by MTT assay. **a** Rb WT and **b** KD MCF-7 cells were treated with a concurrent dosing schedule with different concentrations (5, 10 and 20 μg/mL). Data shown are expressed as mean ± SE of three separate experiments. *p < 0.05 Rib/Eve- vs Rib-treated; #*p* < 0.05 Rib/Eve- vs Eve-treated

**Fig. 4:**
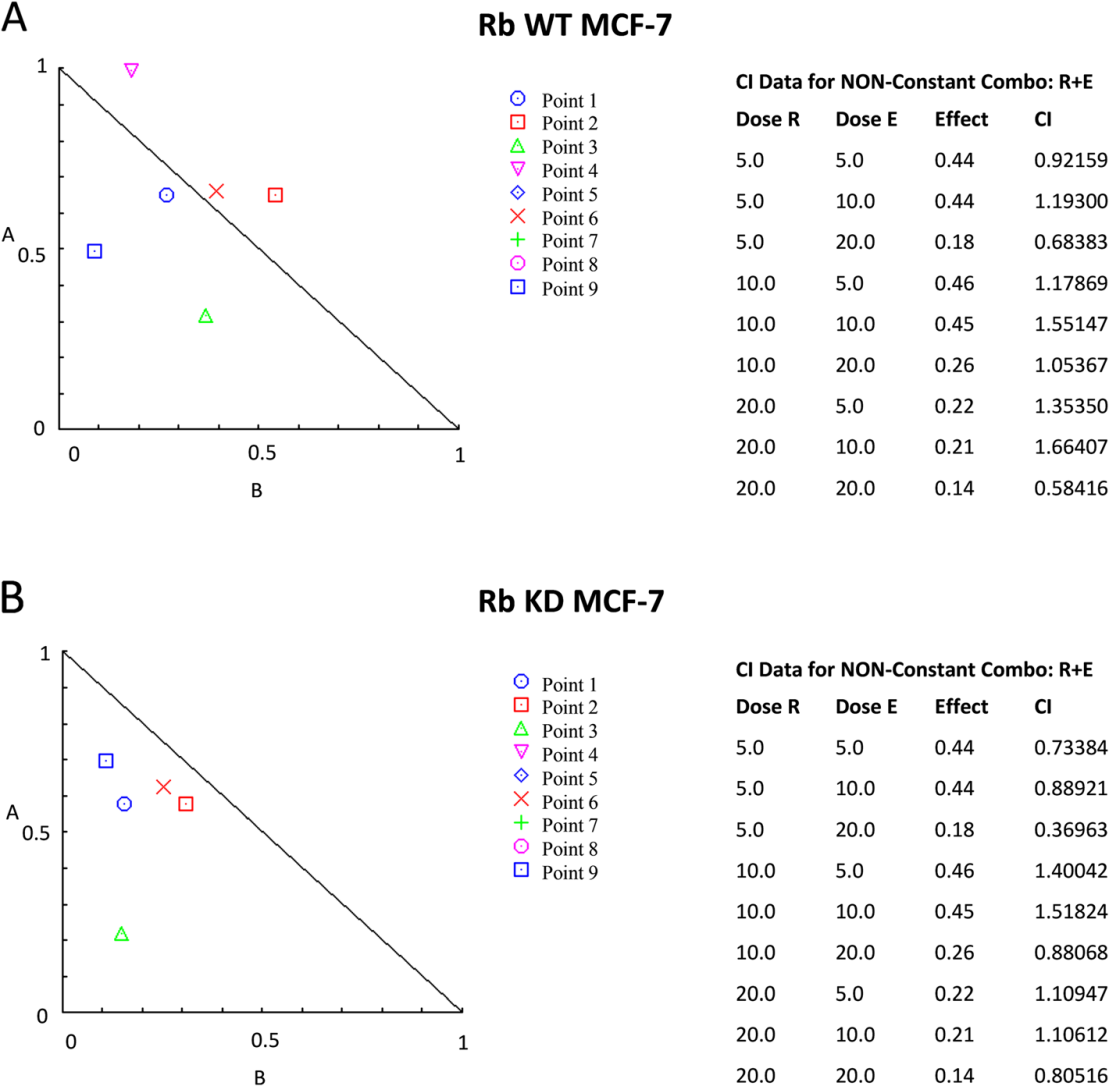
Isobologram plots and CI Values for combination Rib/Eve treatments. On the lower left of the hypotenuse synergism, on the hypotenuse additive effect, and on the upper right of the hypotenuse antagonism. Synergistic activity of Rib/Eve was assessed by CompuSyn software. CI = 1, < 1 and > 1 indicates additive effect, synergism and antagonism, respectively, in Rib/Eve-treated Rb WT (**a**) and KD (**b**) MCF-7 cells

Since no major effects were observed by using Rib/Eve sequential schedule, compared with Rib alone, the effect of concurrent schedule using different doses of Rib/Eve at 72 h after treatment was evaluated (Table 1B). Rib at low dose (5 μg/ml) in combination with Eve at 5 and 20 μg/ml increased the cytotoxicity against Rb WT and KD MCF-7 cells, compared to Rib alone; in addition Rib at 5 μg/ml plus Eve at 10 μg/ml increased the cytotoxicity in Rb KD MCF-7 cells, compared to Rib alone. Moreover, high Rib doses (10 and 20 μg/ml) plus Eve (20 μg/ml) increased the cytotoxicity in Rb WT and KD MCF-7 cell lines, compared to Rib alone (Fig. 3a and b). Rib at all doses increased the Eve-induced cytotoxicity in Rb WT and KD MCF-7 cells, compared to Eve alone. Finally, according to isobologram analysis Rib at 5 μg/ml in combination with Eve at 5 or 20 μg/ml and Rib at 20 μg/ml combined with Eve 20 μg/ml showed a significant synergic effect in Rb WT MCF-7 cells (Fig. 4a). Similarly, in Rb KD MCF-7 cells, Rib at 5 μg/ml in combination with Eve at 5, 10 and 20 μg/ml and Rib at 10 and 20 μg/ml plus Eve 20 μg/ml showed significant synergic effect (Fig. 4b). In addition, additive effect was observed in Rb WT MCF-7 cells by using Rib 10 plus Eve 20 μg/ml (Fig. 4a) and in Rb KD MCF-7 cells by using Rib 20 plus Eve (5 and 10 μg/ml); antagonistic effect was evidenced at the other Rib/Eve drug combinations in both Rb WT and KD MCF-7 cells (Fig. 4a and b).

Thus, in considering of both the effects in sequential schedule at Rib 10/Eve 20 μg/ml drug combination and the results of concurrent schedule, all the other experiments were performed in both WT and KD MCF-7 cells treated with Rib/Eve in combination at 10/15 μg/ml, 1:1.5 ratio.

### Concurrent administration of rib and eve influenced cell cycle in Rb WT and KD MCF-7 cells

**Fig. 5:**
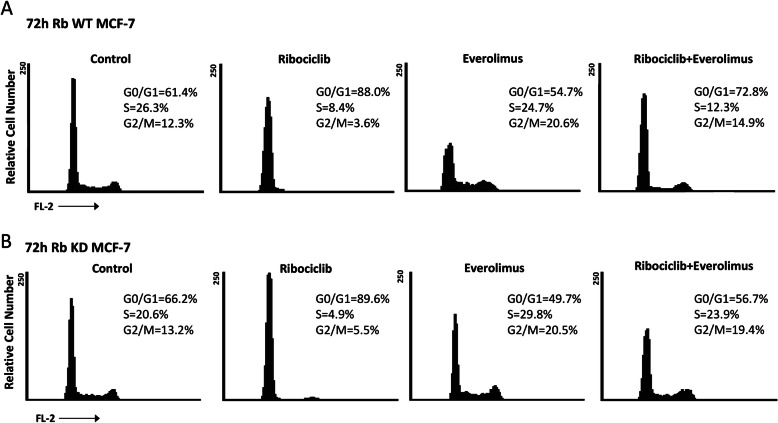
Rib and Eveinfluenced cell cycle in Rb WT and Rb KD MCF-7 cell lines.Rb WT and Rb KD MCF-7 cell lines were treated with Rib and Eve, 10/15 μg/ml, 1:1.5ratio, alone and in combination, for 72 h

Since Rib has been found to arrest BC cells at G1 phase, whereas Eve arrests BC cells at G2/M phase depending of dose [19], we evaluated the effect of Rib/Eve in combination at 72 h post-treatments. Rib alone increased from 61 to 88% the percentage of G1 cell phase and Eve alone increased the percentage of G2/M cells from 12 to 21% in Rb WT MCF-7 cells. Treatment of Rb WT MCF-7 cells with Rib/Eve in combination slightly reduced the percentage of G1 cells from 88 to 73% and G2/M cells from 21 to 15% (Fig. 5a). Similarly, in Rb KD MCF-7 cells, Rib increased the percentage of G1 cells from 66 to 90%. On the other hand, as previously described [19] at the dose used, Eve increased the S and G2/M cell phases from 21 to 30% and from 13 to 20%, respectively (Fig. 5b). No changes in the percentage of G2 cells, with slight decrease of S cells (from 30 to 24%) and increase of G1 cells (from 50 to 57%) were found in Rb KD MCF-7 cells treated with Rib/Eve in combination, suggesting that the Rib-mediated cell cycle control is Rb-dependent.

### Eve increased the rib induced apoptotic effects in Rb WT and KD MCF-7 cells

**Fig. 6:**
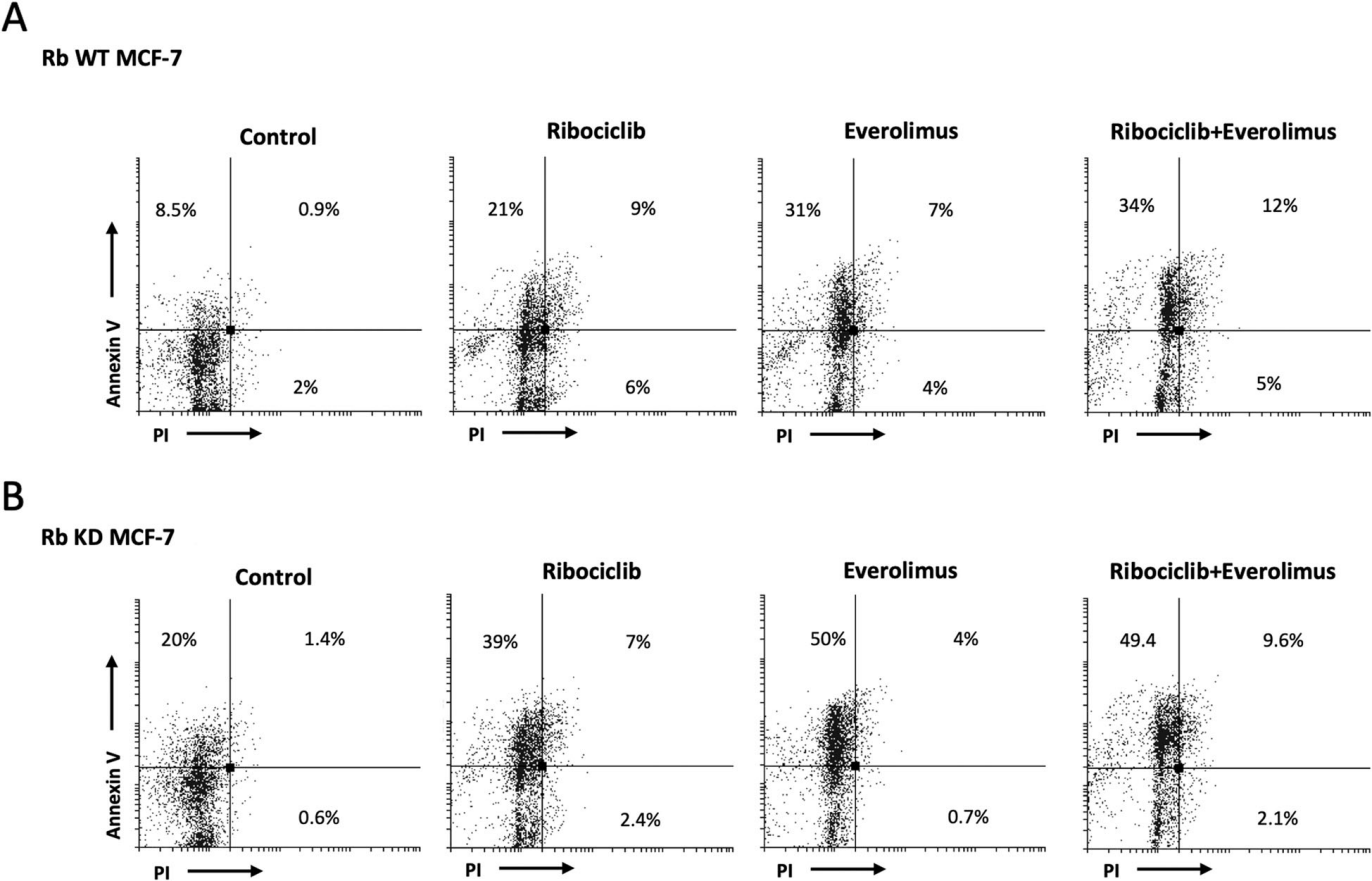
Rib and Eve induced apoptosis in Rb WT and Rb KD MCF-7 cell lines. Rb WT and Rb KD MCF-7 cell lines were treated with Rib and Eve, 10/15 μg/ml, 1:1.5ratio, alone and in combination, for 72 h. Flow cytometry was performed by Annexin V and PI double-staining. Data represent the percentage of PI and/or Annexin V positive cells and are representative of one of three separate experiments

Rib strongly inhibited the proliferation of ER^+^HER2^−^ BC cells, but its effect is predominantly a cytostatic, but not cytotoxic effect. Thus, we evaluated whether Eve administered in combination may increase the Rib effects. In this view we treated both Rb WT and KD MCF-7 cells with the Rib/Eve in combination for 72 h and analyzed cell death by Annexin V-FITC/PI staining and cytofluorimetric analysis. We found that treatment with Rib alone result in 21 and 25% of Annexin V-positive apoptotic cells in Rb WT and KD MCF-7 cells, respectively; similarly, Eve alone induced about 29 and 33% of AnnexinV-positive apoptotic cells (Fig. 6a and b). Concurrent exposure of cells to Rib/Eve resulted in about 37 and 40% of apoptotic cells in Rb WT and KD MCF-7 cells (Fig. 6a and b). Thus, Rib/Eve in combination significantly increases the apoptotic effect from 21 to 37% and from 25 to 40%, compared to Rib alone, in Rb WT and KD MCF-7 cells, respectively.

### Rib induced senescence in Rb WT and Rb KD MCF-7 cells and eve reverted the rib-mediated effect

**Fig. 7:**
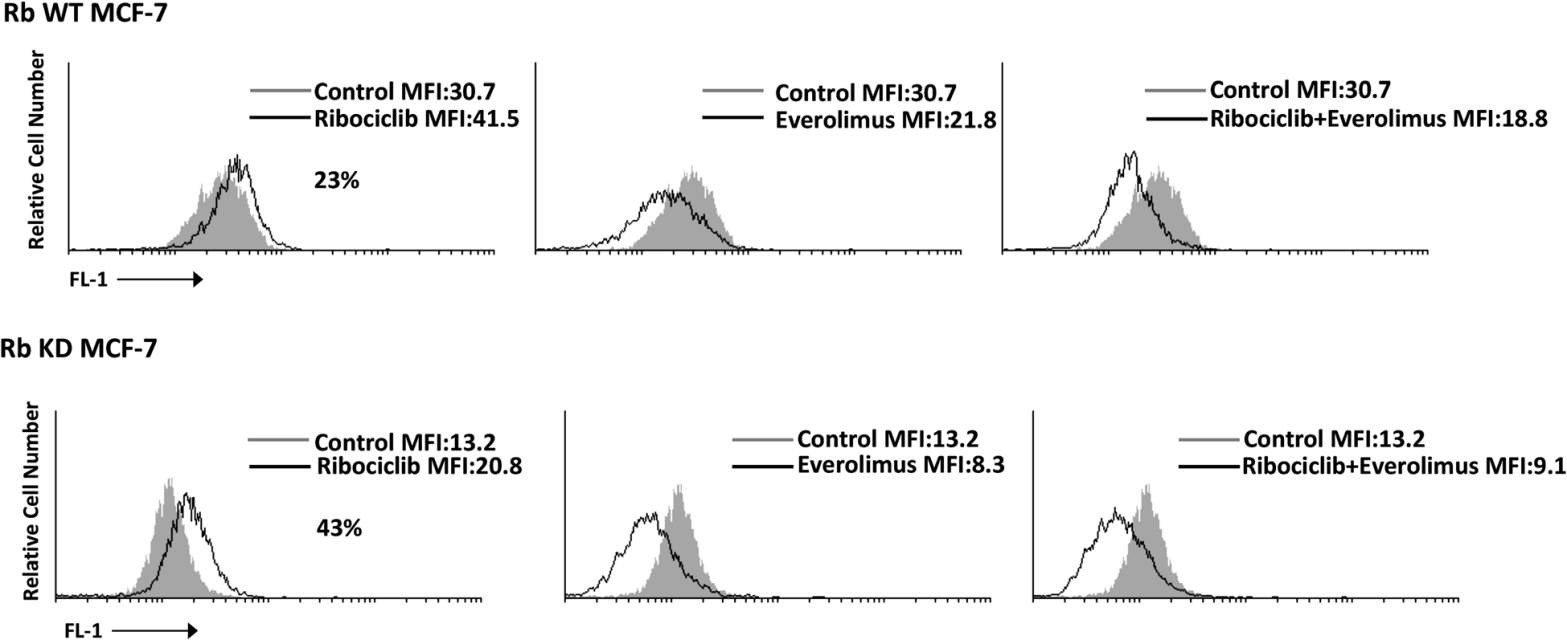
Rib induces senescence in Rb WT and Rb KD MCF-7 cell lines. Rb WT and Rb KD MCF-7 cell lines were treated with Rib and Eve, 10/15 μg/ml, 1:1.5ratio, alone and in combination, for 72 h

Rib has been found to induce cell senescence [4, 25, 26]. Herein, we evaluated the capability of the Eve to affect the Rib-induced senescence of BC cells and the potential role of Rb. Cell senescence was evaluated by cytofluorimetric analysis using the fluorogenic substrate C12FDG both in Rb WT and KD MCF-7 cells, treated for 72 h with Rib or Eve (10/15 μg/ml, 1:1.5ratio), alone or in combination. Thus, about 23% of senescence-associated β-galactosidase (SA-β-Gal^+^) cells were evidenced in Rb WT MCF-7 cells (Fig. 7). No SA-β-Gal+ cells were found in Eve-treated Rb WT MCF-7 cells at 72 h after treatment. In addition, Eve in combination with Rib, completely reverted the Rib-induced cell senescence effect. Treatment of Rb KD MCF-7 cells with Rib increased from 23 to 43%, as compared to Rb WT cells, the percentage of SA-β-Gal^+^ cells compared to Rb WT MCF-7 cells. Similarly, to Rb WT MCF-7 cells, Eve alone did not induce cell senescence in Rb KD MCF-7 cells, and completely reverted the Rib-mediated increase of SA-β-Gal^+^ senescent cells.

Overall, Rib induced cell senescence in MCF-7 cells; down-regulation of Rb enhanced the percentage of senescent cells in MCF-7 cells. Eve did not induce senescence and completely reverted the Rib-mediated effects.

## Discussion

Inhibition of cyclin D-CDK4/6-dependent pathways are emerging as potent anti-cancer strategy for management of hormone receptor HR^+^ and HER2^−^ [9]. Rib is one of the three selective small-molecule inhibitors of the CDK4/6, currently approved for the treatment of the advanced HR^+^ HER2^−^ BC showing better progressive free survival (PFS) outcomes [27]. Increased efficacy of the therapies based on the use of CDK4/6 inhibitor in BCs has been recently obtained by the concomitant use of endocrine therapy. So, high quality of life and increased overall survival (OS), although not significant, were reported in PALOMA-3 (Palbociclib Ongoing Trials in the management of Breast cancer-3) in patients undercome Palbociclib plus endocrine therapy than those with endocrine therapy alone [27]. Recent results of MONALEESA-7 (Mammary Oncology assessment of LEE011’s Efficacy and Safety 7) showed significant OS with Rib plus endocrine therapy, than endocrine therapy alone among premenopausal or perimenopausal HR^+^ HER2^−^ advanced BC patients [28, 29]. Finally, a significant improvement in OS with the Abemaciclib plus the endocrine therapy compound Fulvestran, compared to endocrine therapy alone, in the MONARCH 2 trial has been reported [30]. Although the CDK4/6 inhibitors in combination with the endocrine therapy have increase OS and PFS, the mortality and adverse effect of 3/4 are frequent, taking into account these clinical results, there is the need of new combination therapies in order to ameliorate OS and/or PFS benefit and also the time to second decrease progression, time to chemotherapy and chemotherapy free survival.

The CDK4/6 kinase is part of the cyclin D/CDK4/6/Rb/E2F1 pathway controlling the cell cycle progression. Based on the mechanism of action, the CDK4/6 inhibitors are predicted to arrest cells in G1 phase resulting in cytostatic effects [2, 9], while Eve, a mTOR inhibitor, arrested cells both in G1 and in G2/M phases, depending of dose, inducing cytostatic or cytotoxic effects [19, 20]. Inhibition of either mTOR signaling or CDK/Rb/E2F signaling causes a decrease in E2F-dependent transcription and the expression of genes required for S-phase entry. Optimal inhibition of Rb/E2F-mediated transcription is only achieved upon blockade of both pathways and the convergent effects of multiple pathway inhibition in repressing Rb/E2F activity is likely to be required to achieve sustained and durable growth [3]. Due to the distinct phases of the cell cycle at which these two class of drugs act, a sequential or concurrent dosing schedule is suggested to achieve enhanced cytotoxicity [3].

Herein, we found that Rib alone strongly reduced the viability of Rb WT MCF-7 BC cells at micromolecular dose. The sequential schedule with Rib for 72 h followed by Eve for 48 h and vice versa did not induce significative increase of cytotoxic activity, compared to Rib alone, except for Rib/Eve10/20 μg/ml in combination. Conversely, the sequential administration of Eve, followed by Rib, markedly increased the Eve-mediated effects in Rb WT MCF-7 cells. The Eve/Rib sequential schedule was more effective than Rib/Eve in Rb WT BC. Thus, cells arrested in S and G2/M phases, that partially repair the damage induced by Eve, will enter the G1 phase where they may become susceptible to Rib inhibition. Moreover, the presence of partially repaired DNA damage from prior exposure to Eve may increase the rate of cells in G1 arrest due to Rib which is able to induce cell senescence.

Since no major effects were observed by using Rib/Eve sequential schedule, compared with Rib alone, the effect of concurrent schedule using different doses of Rib/Eve in combination was evaluated. The Rib/Eve combination significantly synergizes to reduce the viability of Rb WT MCF-7 cells.

Further analysis on the Rb-dependent effects of the concurrent use of Rib/Eve compared to Rib alone, evidenced that it maximally arrested Rb WT MCF-7 cells at G1 phase (about 90%), but only a 21% of cells underwent apoptotic cell death, supporting its more evident cytostatic activity. Moreover, about 23% of Rb WT MCF-7 cells showed a senescent phenotype. Eve, arresting Rb WT MCF-7 cells at G2/M phase (21%), did not affect cell proliferation, induced apoptosis (about 31% of cells) and resulted in a complete drop of SA-β-gal^+^ senescent cell population. The Rib/Eve combination moderately reduced the percentage of cells in G1 and G2/M phases (from 88 to 73% and 20 to 15%, respectively), double the percentage of Annexin V-positive apoptotic cells, compared to that triggered by Rib alone (from 21 to 34%), and completely reverted the Rib-induced senescence of Rb WT MCF-7 cells. Regarding the effects of Eve on cell cycle progression it has been demonstrated a dose-dependent effect. Thus, at high doses, in the same range of that used to us, it increases pRb, cyclin D1, Ki67 and prompted transition from G2 cells into mitosis [19], rather than stable senescence [4, 25, 26].

Senescence is a state of irreversible cell growth arrest and metabolic activity maintenance, driven by TP53, CDKN1A/p21, CDKN1B/p27 and CDKN2A/INK4/ARF proteins. It acts both as endogenous antitumor mechanism by avoiding the proliferation of transformed precancerous cells [31, 32], but also it has been associated with a phenotype of apoptosis-resistant cancer cells [33, 34]. Our results display that the Rib-mediated senescence in Rb WT BC cells are reverted by concurrent Eve treatment. These data are in agreement with recent reports showing that differently from classical senescence that is irreversible, senescence induced by CDK4/6 inhibitors (Palbociclib, Rib) may be reversible, suggesting that it may be a senescent-like quiescence. It may depend to slow release of these drugs from lysosomes or proteasome hyperactivation [35].

In addition, Rib, reducing Rb phosphorylation, leads to suppression of mTORC2 activation [36] and increased mTORC1 activity [37]. Eve is a rapalogs such as rapamycin; it is a gero-suppressant, which decreases cellular senescence. Moreover, Eve is an inhibitor of mTORC1 activity [38]. Inhibition of both mTORC1 and mTORC2 activity seems to be necessary to elicit maximal cellular changes required to reverse senescence phenotype [39]. Thus, although Eve does not induce senescence, it inhibits mTORC1 and when administered with Rib that inhibits mTORC2, reverts cell senescence. In this regard, it has been reported that the dual mTORC1/2 inhibitors Vistusertib, or Eve, in concurrent combination with Palbociclib in HR+ BC, showed a decrease in the levels of Rb phosphorylation, more than single agent [3].

Rb is a tumor suppressor that regulates late G1 restriction point, DNA damage response checkpoints, cell cycle exit and differentiation [40]. The Rb pathway (INK4-cyclin D-cdk4/6-Rb) controls the G1-S phase transition; Cyclin D-CDK4/6 complexes initiate G1 progression by phosphorylating (inactivating) Rb, thus relieving transcriptional repression by the Rb-E2F complex. Following Rb phosphorylation, E2F is released, inducing transcription of genes necessary for S-phase entry. The hyperphosphorylation of Rb reduces the affinity for E2F making possible cell division. The effect of CDK4/6 inhibitors on inhibiting tumor cell growth is achieved by blocking the phosphorylation of Rb in a low nano/micromolar range [40]. CDK4/6 inhibitors have been demonstrated to be effective against a variety of Rb-positive tumors, including BC, however most Rb-negative tumor cells are resistant to CDK4/6 inhibitors. Direct analysis of primary tumors report loss of Rb function in 20 to 35% of BCs [41]. Moreover, in BC cell lines, chronic loss of Rb has been associated with the development of CDk4/6 inhibitor-resistance state [42]. However, the prevalence of RB1 mutations in endocrine-pretreated and CDK4/6 inhibitor resistant BC is unknown. In PALOMA-3 trial has been demonstrated that RB1 mutation emerged in a minority, about 5% of patients treated with Palbociclib plus Fulvestrant as a consequence of therapy pressure [43]. Moreover, Rib may have different level of selectivity for other CDKs other than CDK4/6 kinase, and it has been hypothesized that such differences could potentially have clinical relevance [44, 45].

Thus, the Rb-independent effects of different doses of Rib or Eve alone or in combination (concurrent schedule) have been also evaluated. Rib reduced the viability of Rb KD MCF-7 cells, although at less extension (3-fold less), compared to Rb WT BC cells (IC_50_ at 48 h: 17 vs 6 μg/ml). No major effects on BC cells were observed by sequential administration of Rib/Eve and vice versa, whereas concurrent administration of Eve at any dose tested, to Rib 5/10 μg/ml increased the Rib anti-proliferative effects. As demonstrated in Rb WT BC cells, although Rib or Eve alone increased the percentage of G1 cells (from 66 to 90%) and G2 cells (from 13 to 20%). In addition, Eve increased the percentage of cells in S phase (from 21 to 30%), likely as consequence to a specific loss of cells undergoing mitosis [19]. Rib/Eve in combination reverted the Rib-induced G1 arrest (from 90 to 57%), while no significative changes in S and G2/M cell phases was reported in Rb KD MCF-7 cells. Rib/Eve in combination increased the percentage of apoptotic cells from 26 to 40% in Rb KD MCF-7 cells, compared to Rib alone, and the percentage of SA-β-gal^+^ senescent cells, from Rib-treated Rb WT (23%) to Rb KD (43%) MCF-7 cells. The increase of cell senescence in Rb KD, compared to Rb WT BC cells, may be the result of Rb depletion. In fact, has been reported that inhibition of Rb phosphorylation leads to increased mTORC2 activation [36] and mTORC2 activation promotes cell senescence [46]. Finally, Eve induced the 33% of apoptosis and no cell senescence, but reverted the Rib-induced senescence in Rb KD MCF-7 cells.

In regard to the Rb-independent effects, has been also reported that the CDK4/6 inhibitor Palbociclib induces a significant cell cycle arrest in Rb-silenced HepG2 and Hu7 hepatocellular carcinoma cell lines [47], as well as induces apoptosis of T-acute lymphoblastic leukaemia [48] and bladder cancer cells [49].

It has been demonstrated that cancer cell senescence induced by CDK4/6 inhibition can promote the development of more aggressive drug-resistant cancer stem cells [50–52] leading to attenuated response to anti-tumor agents and cancer recurrence. Thus, it can be hypothesized that Eve, by reverting the senescence induced by Rib in both MCF-7models, may abrogate/reduce the development of BC stem cells, thus avoiding the consequent drug-resistance.

Knock-down of Rb in MCF-7 cells does not abrogate cell senescence. The p53/p21Cip1 and Rb/p16 pathways represent the major inductors of cell senescence, thus in Rb KD/p16-mutated MCF-7 cells the p53/p21 signaling pathway trigger the senescence of BC cells [53].

Another advantage for the use of concurrent therapy with Rib/Eve has been just evidenced in triple joint therapy of Rib with Exemestane and Eve in HR^+^ ABC patients. It has been reported that after the combined use of Rib, the level of Eve metabolized by cytochrome P450 (CYP3A4) increased by 1.5 to 3-fold, with the possibility to reduce the Eve-mediated drug toxicity [9]. On the contrary the use of Rib/Eve in combination reduced the Rib induced adverse effects. The most common effects of CDK4/6 inhibitors are neutropenia, leucopenia, fatigue, nausea [6] and cardiotoxicity [54]. Moreover, since Rib is orally taken, and its metabolism could be influenced by CYP3A enzyme, the increased toxicity may also result by interaction with CYP3A inhibitors or CYP3A inducers [55].

The combination of CDK4/6 and mTOR inhibitors will produce more effects on regression of cell growth by increasing the cytotoxicity in both Rb WT and KD MCF-7 cells, which overcome the resistance to the sole use of CDK4/6 inhibitors and delay initiation of chemotherapy [3, 56].

Overall, combination of a CDK4/6 inhibitor, Rib and the mTOR inhibitor, Eve may represent a promising strategy for HR^+^ HER2- BC patients, also in Rb-deficient tumors.

## Conclusions

In synthesis the use of Rib/Eve in concurrent therapy, compared to monotherapy with Rib or Eve alone or sequential therapy with Rib/Eve schedule or vice versa, results in several advantages: a) increases the pro-apoptotic effect compared to that of Rib alone b) decreases the levels of Rb phosphorylation and consequently augment the efficiency of treatment c) inhibits the Rib-induced senescence and avoids the BC stem-cells generation and apoptotic resistance d) avoids the Rib-induced resistance by reducing Rib dose and delays the initiation of chemotherapy.

Whether the data presented in in vitro setting hold true in vivo, should require further study and clinical investigation.

## Supplementary Information

**Additional file 1: Figure S1.** Rb silencing in MCF-7 cells. Western blot analysis of Rb and β-actin protein levels in MCF-7 BC cells. β-actin protein levels were evaluated as loading control. Blots are representative of one of three separate experiments. Bars represent the densitometric analysis. *p < 0.05 Rb WT MCF-7 vs Rb KD MCF-7.

**Additional file 2: Figure S2.** Rib induced cytotoxicity in BT-549 cell line. Cell viability was determined by MTT assay. BT-549 cells were treated for A) 48 hs and B) 72 hs with different concentrations of Ribociclib. Data shown are expressed as mean ± SE of three separate experiments. *p < 0.05 treated vs untreated.

## Data Availability

All relevant data have been provided in the text and on-line supplement. Data Sharing: data are available from the corresponding author at giorgio.santoni@unicam.it.

## Abbreviations

ABC: Advanced Breast Cancer
BC: Breast cancer
CDK: Cyclin-Dependent Kinase
ER +: Estrogen Receptor positive
Eve: Everolimus
HR +: Hormone Receptor-positive
HER2-: Human Epidermal growth factor Receptor 2-negative
mTORC1: mTOR complex 1
mTORC2: mTOR complex 2
OS: overall survival
pRb: Phosphorilation of Retinoblastoma Protein
PFS: progressive free survival
KD: Rb knockdown
WT: Rb wild type
Rb: Retinoblastoma gene
Rib: Ribociclib
SA-β-Gal+: Senescence-Associated β-galactosidase
siRNAs: Small interfering RNAs
