# Supplementary figures and images for: Exploring treatment with Ribociclib alone or in sequence/combination with Everolimus in ER+HER2−Rb wild-type and knock-down in breast cancer cell lines
Source: BMC Cancer. 2020 Nov 19;20:1119. doi: 10.1186/s12885-020-07619-1 (PMC7678099; doi:10.1186/s12885-020-07619-1)

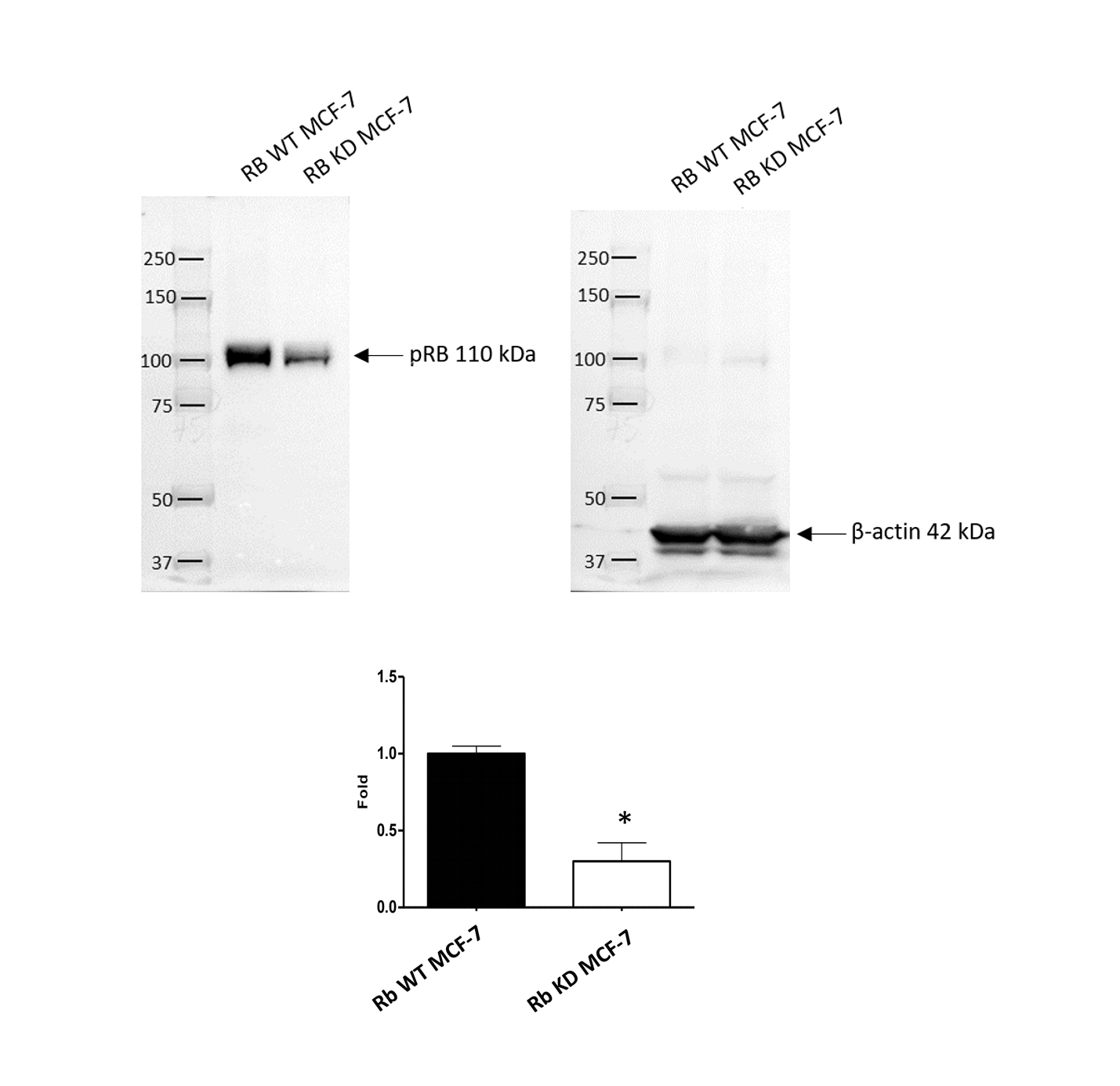

Supplement: Supplementary file 1 — Additional file 1: Figure S1. Rb silencing in MCF-7 cells. Western blot analysis of Rb and β-actin protein levels in MCF-7 BC cells. β-actin protein levels were evaluated as loading control. Blots are representative of one of three separate experiments. Bars represent the densitometric analysis. *p < 0.05 Rb WT MCF-7 vs Rb KD MCF-7. [file 12885_2020_7619_MOESM1_ESM.tif]

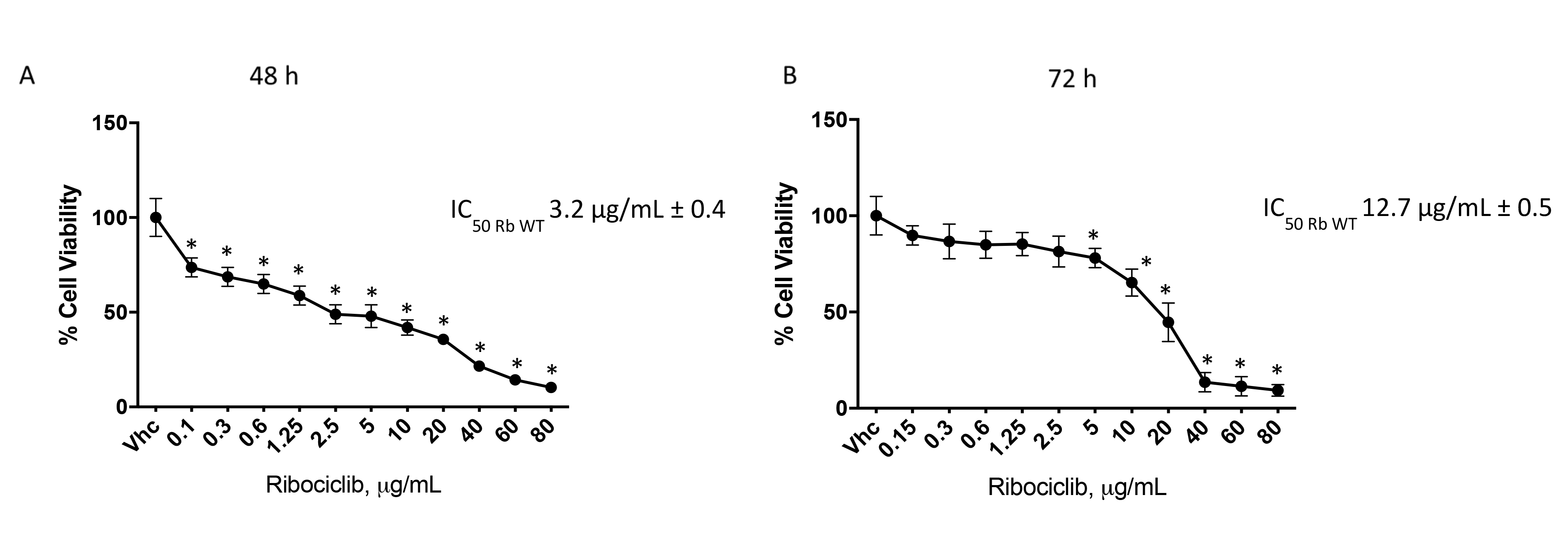

Supplement: Supplementary file 2 — Additional file 2: Figure S2. Rib induced cytotoxicity in BT-549 cell line. Cell viability was determined by MTT assay. BT-549 cells were treated for A) 48 hs and B) 72 hs with different concentrations of Ribociclib. Data shown are expressed as mean ± SE of three separate experiments. *p < 0.05 treated vs untreated. [file 12885_2020_7619_MOESM2_ESM.tif]
